# Autologous antigen-presenting cells efficiently expand *piggyBac* transposon CAR-T cells with predominant memory phenotype

**DOI:** 10.1016/j.omtm.2021.03.011

**Published:** 2021-03-23

**Authors:** Kayoko Nakamura, Shigeki Yagyu, Shogo Hirota, Akimasa Tomida, Makoto Kondo, Tomokuni Shigeura, Aiko Hasegawa, Miyuki Tanaka, Yozo Nakazawa

**Affiliations:** 1 Department of Pediatrics, Shinshu University School of Medicine, 3-1-1 Asahi, Matsumoto, Nagano, Japan; 2 Department of Pediatrics, Kyoto Prefectural University of Medicine, Graduate School of Medical Science, 465 Kajii-cho, Kawaramachihirokoji, Kamigyo-ku, Kyoto, Japan; 3 Center for Advanced Research of Gene and Cell Therapy, Shinshu University School of Medicine, 3-1-1 Asahi, Matsumoto, Nagano, Japan; 4 Department of Pharmaceutical Development, BrightPath Biotherapeutics, Co., Ltd., 2-2-4 Kojimachi, Chiyoda-ku, Tokyo, Japan; 5 Department of Cell Therapy, BrightPath Biotherapeutics, Co., Ltd., 2-2-4 Kojimachi, Chiyoda-ku, Tokyo, Japan; 6 Institute for Biomedical Sciences, Interdisciplinary Cluster for Cutting Edge Research, Shinshu University, 3-1-1 Asahi, Matsumoto, Nagano, Japan

**Keywords:** chimeric antigen receptor T cells, piggyBac transposon, HER2, early T cell exhaustion, T stem cell memory-like cells, electroporation

## Abstract

The quality of chimeric antigen receptor (CAR)-T cell products, including the expression of memory and exhaustion markers, has been shown to influence their long-term functionality. The manufacturing process of CAR-T cells should be optimized to prevent early T cell exhaustion during expansion. Activation of T cells by monoclonal antibodies is a critical step for T cell expansion, which may sometimes induce excess stimulation and exhaustion of T cells. Given that *piggyBac* transposon (PB)-based gene transfer could circumvent the conventional pre-activation of T cells, we established a manufacturing method of PB-mediated HER2-specific CAR-T cells (PB-HER2-CAR-T cells) that maintains their memory phenotype without early T cell exhaustion. Through stimulation of CAR-transduced T cells with autologous peripheral blood mononuclear cell-derived feeder cells expressing both truncated HER2, CD80, and 4-1BBL proteins, we could effectively propagate memory-rich, PD-1-negative PB-HER2-CAR-T cells. PB-HER2-CAR-T cells demonstrated sustained antitumor efficacy *in vitro* and debulked the HER2-positive tumors *in vivo*. Mice treated with PB-HER2-CAR-T cells rejected the second tumor establishment owing to the *in vivo* expansion of PB-HER2-CAR-T cells. Our simple and effective manufacturing process using PB system and genetically modified donor-derived feeder cells is a promising strategy for the use of PB-CAR-T cell therapy.

## Graphical abstract

**Figure:**
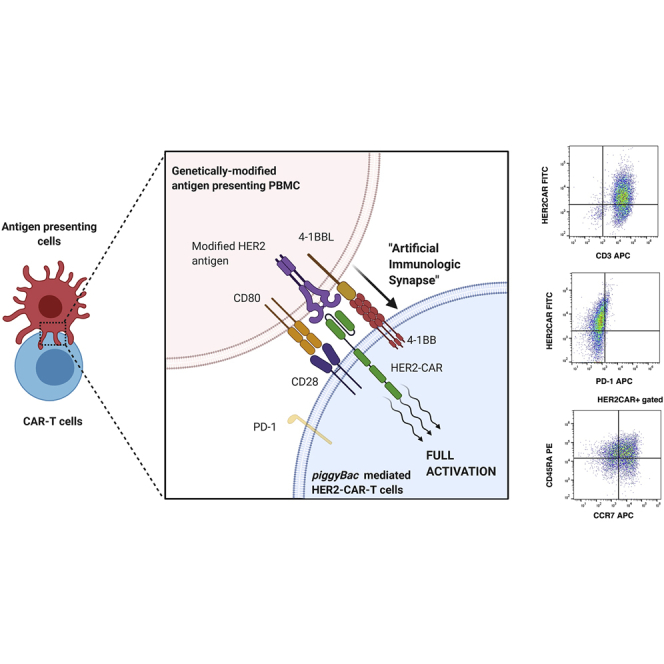

## Teaser

We developed a clinically applicable method for manufacturing various *piggyBac* transposon (PB)-mediated HER2-CAR-T cells that exhibit minimal T cell exhaustion profiles, leading to sustained tumor control. Our manufacturing process has the potential for producing various CAR-T cells enriched with the memory phenotype associated with stable antitumor efficacy for clinical use.

## Introduction

Recent clinical trials on chimeric antigen receptor (CAR)-T cell therapy have demonstrated that the quality of CAR-T cell products, including the expression of immune exhaustion markers, determined their function and antitumor efficacy.^1^^,^^2^ In particular, early T cell exhaustion during the manufacturing process is one of the key causes of impairment of the quality and function of CAR-T cells, evidenced by low proliferative and cytokine-producing capacities, high rates of apoptosis, and high expression of inhibitory receptors such as programmed cell death receptor (PD-1).^3^ Therefore, the manufacturing process of CAR-T cells should be optimized to prevent early T cell exhaustion and maintain their memory phenotype during the expansion step.

Non-viral gene transfer, including *Sleeping Beauty* (SB) or *piggyBac* (PB) transposon-based genetic modifications, is a potentially effective strategy to manufacture CAR-T cells,^4^, ^5^, ^6^, ^7^, ^8^, ^9^, ^10^ since transposon enables stable expression of the genes of interest when the genes and transposase are introduced into either pre-activated or resting T cells. However, drawbacks of this approach include low transduction efficiency and cell expansion capacity.^5^^,^^11^ Co-culture of T cells with anti-CD3 and -CD28-specific monoclonal antibodies has been well established for the activation and expansion of T cells;^12^ nevertheless, unoptimized, excessive activation of T cells by monoclonal antibodies could lead to terminal differentiation or activation-induced cell death, especially for electroporated T cells.^13^ Instead, feeder cells were used to expand T cells and genetically engineered K562 cell lines that expressed various factors, including a specific antigen or anti-CD3 antibody, co-stimulatory molecules, cytokines, and suicide gene systems and that have been extensively analyzed for the expansion of T cells;^9^^,^^14^ however, the potential risk of contamination of the tumor cells is a considerable safety concern. Researchers have also attempted to use unmanipulated peripheral blood mononuclear cell (PBMC)-derived feeder cells for the expansion of CD19-CAR-T cells, which requires a relatively long expansion.^13^^,^^15^ Furthermore, the procedures for manufacturing various PB-CAR-T cells other than CD19-CAR-T cells are underexplored.

Optimal T cell activation requires a formation of immunological synapse that initiates proliferation, effector function, or death, depending on the intensity of the T cell receptor (TCR) signal and associated signals. The activation of TCR without co-stimulation results in T cell unresponsiveness, anergy, or exhaustion.^16^ Based on this evidence, we hypothesize that the physiological interaction of PB-CAR-T cells and autologous PBMC-derived genetically engineered antigen-presenting feeder cells through the “artificial immunological synapse,” incorporating both specific antigen stimulation and ample co-stimulation, would optimally stimulate PB-CAR-T cells without inducing early T cell exhaustion. In this study, using HER2-specific CAR-T cells as a model, we aimed to develop a clinically applicable method for manufacturing various PB-CAR-T cells that exhibit a lower exhaustion profile, since they are a promising and realistic therapeutic option for targeting refractory tumors.

## Results

### Stimulation of CAR-T cells by antigen-expressing tumor cells and/or anti-CD3/CD28 antibodies did not improve expansion of CAR-T cells

**Figure 1:**
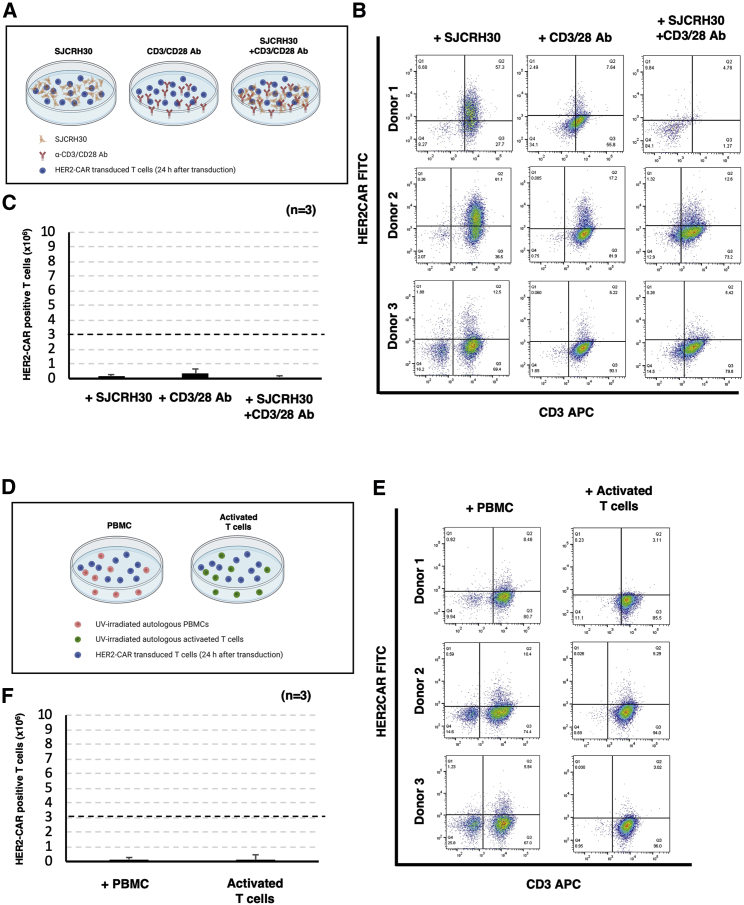
Stimulation of CAR-T cells by antigen-expressing tumor cells, anti-CD3/CD28 antibody, or autologous PBMCs does not enhance the expansion of PB-HER2-CAR-T cells (A) Schematics of the stimulation of CAR-transduced T cells with tumor cells or antibody. We transduced pIRII-HER2-CAR plasmid encoding HER2-CAR transgene with pCMV-PB plasmid encoding PB transposase into resting PBMC using electroporation. After 24 h, we collected approximately 3 million live cells, which were subsequently stimulated with 3 million UV-irradiated HER2-positive SJCRH30 tumor cells (SJCRH30), plate-bound anti-CD3/CD28 monoclonal antibodies (CD3/CD28 Ab), or both (SJCRH30+CD3/CD28 Ab). (B) Representative dot plots of flow cytometry analysis for the expression of HER2-CAR and CD3 in each condition. (C) Absolute number of CAR-positive T cells at day 14. The mean ± SD from 3 donors are shown. Dot line indicates the initial number of the live cells on day 1. (D) Schematics of the stimulation of CAR-transduced T cells with autologous PBMCs (PBMC) or autologous activated T cells (activated T cells). (E) Representative dot plots of flow cytometry analysis for the expression of HER2-CAR and CD3 in each condition. (F) The number of CAR-positive T cells at day 14. The mean ± SD from 3 donors are shown. Dot line indicates the initial number of the live cells on day 1.

Initially, we evaluated the effect of the stimulation by HER2-positive tumor cells or anti-CD3/CD28 antibodies for the expansion of PB-HER2-CAR-T cells. We transduced pIRII-HER2-CAR plasmid encoding HER2-CAR transgene (Figure S1) with pCMV-PB plasmid encoding PB transposase into fresh, unstimulated PBMC by electroporation. The transduction efficacy of the CAR transgene 24 h after electroporation was 24.6% ± 12.9% (Figure S2). Approximately 3 million live cells were collected 24 h after electroporation and were stimulated with the same amount of UV-irradiated HER2-positive SJCRH30 tumor cells, through plate-bound anti-CD3/CD28 monoclonal antibodies, or both for 48 h, and maintained in complete culture medium (CCM) for 14 days (Figure 1A). Stimulation of CAR-transduced T cells with HER2-positive tumor cells and/or plate-bound anti-CD3/CD28 monoclonal antibodies did not improve the expansion of PB-HER2-CAR-T cells, although we observed HER2-CAR-positive T cell enrichment in the SJCRH30 stimulation group (Figures 1B and 1C). In addition, we attempted the stimulation of HER2-CAR-transduced T cells with UV-irradiated, unmanipulated autologous PBMC or UV-irradiated autologous pre-activated T cells with anti-CD3/CD28 antibodies 24 h after electroporation, as previously established for the production of PB-CD19-CAR-T cells^13^^,^^15^ (Figure 1D), but we did not observe any supportive effects on the expansion of PB-HER2-CAR-T cells (Figures 1E and 1F).

### Stimulation of CAR-T cells using genetically modified autologous PBMCs expressing modified antigen and co-stimulatory molecules greatly enhanced the expression of CAR-T cells with stem cell memory-like phenotype

**Figure 2:**
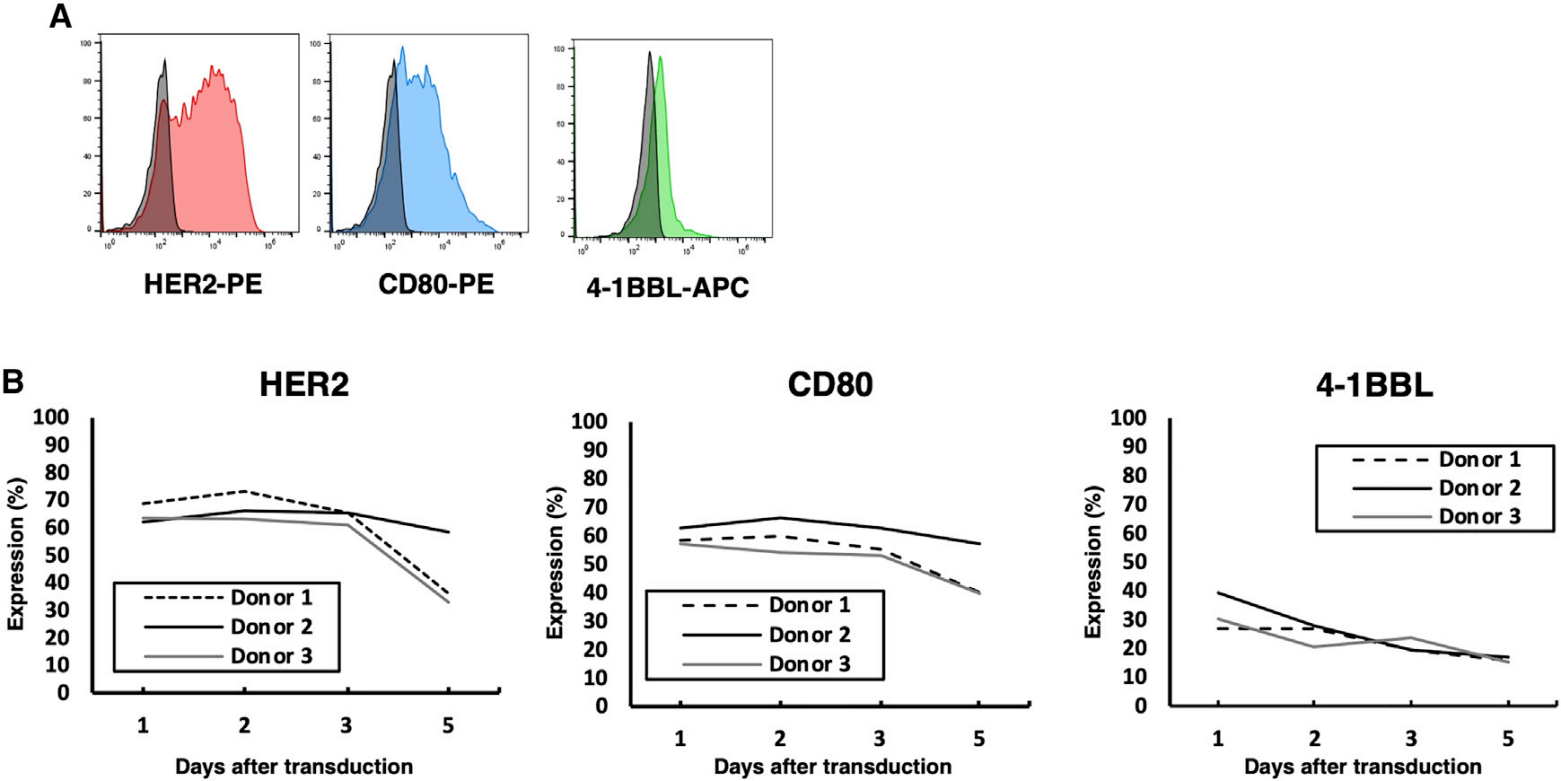
Manufacturing of autologous PBMC-derived antigen-presenting feeder cells (A) Expression of truncated HER2, CD80, and 4-1BBL on the cell surface of antigen-presenting feeder cells 24 h post-electroporation. (B) Percentage of truncated HER2, CD80, and 4-1BBL-positive cells over time for 5 days after transduction.

**Figure 3:**
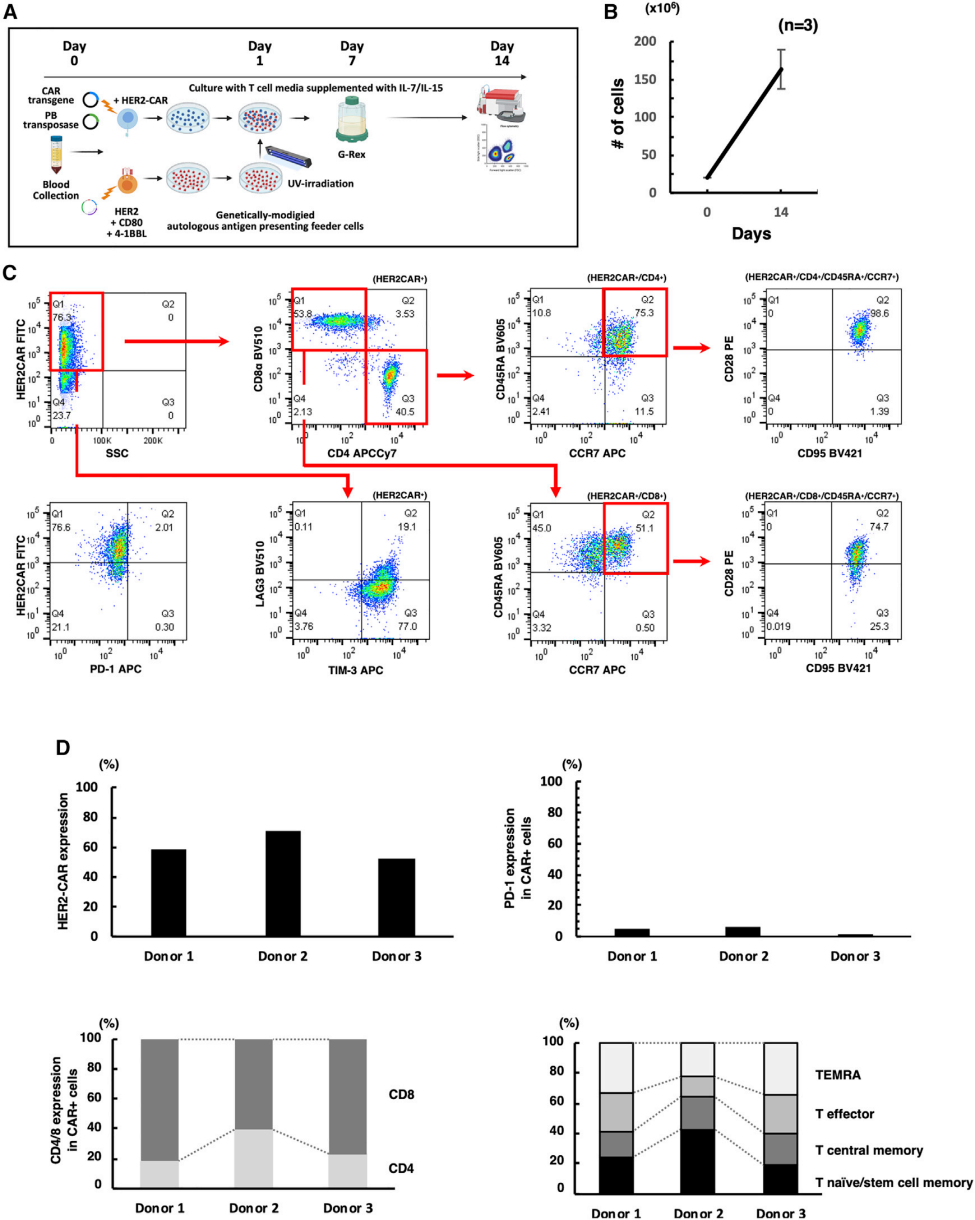
Manufacturing and characteristics of PB-HER2-CAR-T cells stimulated with autologous PBMC-derived antigen-presenting feeder cells (A) Schematics of the manufacturing procedure of PB-HER2-CAR-T cells. (B) Absolute numbers of PB-HER2-CAR-T cells after 14 days of culture. (C) Representative characteristics of PB-HER2-CAR-T cells on day 14. (D) Phenotype of PB-HER2-CAR-T cells regarding CAR expression, PD-1 expression, CD4/CD8 ratio, and differentiation profiles in HER2-CAR-T cells established from three different donors. TEMRA; effector memory T cells re-expressing CD45RA.

We hypothesized that the stimulation of T cells by antigen-presenting feeder cells through physiological immunological synapses involving clustering of antigen-CAR interaction and co-stimulatory molecules would optimally activate PB-HER2-CAR-T cells after electroporation. To prove this, we created autologous feeder cells that express antigen/co-stimulatory molecules by introducing pIRII-tHER2-CD80-4-1BBL on autologous PBMCs to overexpress truncated HER2, CD80, and 4-1BBL (Figure S1). The expression of truncated HER2, CD80, and 4-1BBL 24 h after electroporation was determined to be 43.0% ± 9.4%, 36.3% ± 9.7%, and 29.7% ± 7.7%, respectively (Figure 2A), and gradually decreased over time for 5 days (Figure 2B), although the expression of endogenous HER2, CD80, and 4-1BBL on unmanipulated PBMCs was at the basal level (Figure S3). The majority of the tHER2-positive feeder cells were CD3 positive, while a small proportion of tHER2-positive cells were CD3-negative/CD56-positive natural killer (NK) cells and CD3-negative/CD19-positive B cells (Figure S4). When we cultured HER2-CAR-transduced T cells with the same amount of UV-irradiated antigen-presenting feeder cells on day 1 (Figure 3A), the expansion and positivity of CAR-positive T cells were greatly enhanced, with an expansion of 8.6 ± 1.1-fold (Figure 3B) and CAR positivity of 60.5% ± 9.0% (Figures 3C and 3D) on day 14. We also assessed the phenotype of PB-HER2-CAR-T cells in terms of CD4/CD8 ratio, the expression of exhaustion markers, and memory/effector phenotype using flow cytometry. PB-HER2-CAR-T cells tended to skew CD8-positive (Figures 3C and 3D). PD-1 was expressed at very low levels in PB-HER2-CAR-T cells on day 14 (4.0% ± 2.3% in CAR-positive T cells), though other activation/exhaustion markers, TIM-3 and LAG3, were modestly expressed (Figures 3C and S5A). CD45RA^+^/CCR7^+^/CD28^+^/CD95^+^ T stem cell memory-like phenotype was dominant in the final product of PB-HER2-CAR-T cells (Figures 3C and S5B), which is a favorable CAR-T cell phenotype,^1^ and this tendency was similarly observed in CD4-positive or CD8-positive subpopulations (Figure S5C). To further assess the influence of co-stimulation of the antigen-presenting feeder cells on CAR-T cell phenotype, we stimulated HER2-CAR transduced T cells with the autologous PBMC-derived feeder cells expressing only truncated HER2 on day 1 and evaluated the phenotype of PB-HER2-CAR-T cells on day 14. PB-HER2-CAR-T cells stimulated by the autologous PBMC-derived feeder cells expressing only truncated HER2 showed a smaller T stem cell memory-like population than those expanded by the feeder cells expressing tHER2, CD80, and 4-1BBL (Figure S6A and S6B) and failed to control the tumor growth in the co-culture assay (Figure S6C). These data indicate the necessity of co-stimulation for the enrichment of memory CAR-T cells.

We also produced antigen-presenting feeder cells for PB-CD19-CAR-T cells by introducing truncated CD19, CD80, and 4-1BBL genes simultaneously into PBMCs. By stimulating CD19-CAR transduced T cells with the feeder cells once on day 1, we obtained > 80% CD19-CAR-positive T cells with > 10-fold expansion in 14 days of culture (Figure S7). The PB-CD19-CAR-T cells also exhibited a dominant T naive/stem cell memory-like cell fraction with scarce expression of PD-1 (Figure S7). Of note, this approach could also be applied to manufacture PB-mediated GD2-specific CAR-T cells (PB-GD2-CAR-T cells) by introducing GD2 and GD3 synthase for the induction of GD2 expression in PBMCs, together with CD80 and 4-1BBL (Figures S8A and S8B), which indicates the versatility of this manufacturing concept to induce memory-phenotype enriched PB-CAR-T cells.

### PB-HER2-CAR-T cells showed robust and sustained antitumor activity against HER2-positive tumors *in vitro*

**Figure 4:**
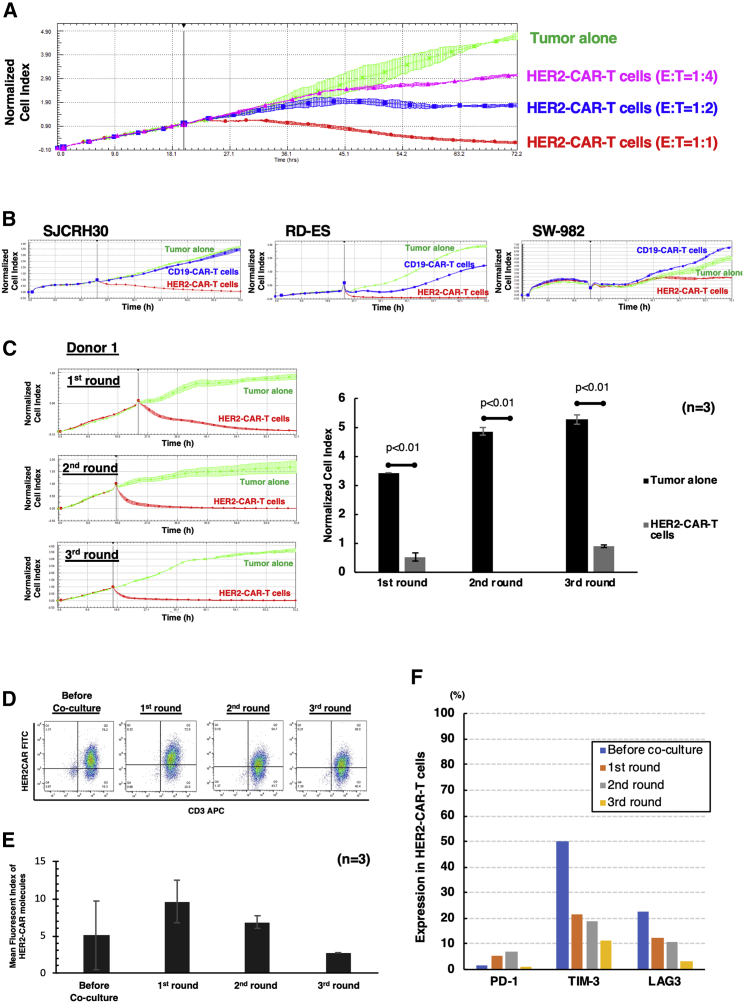
PB-HER2-CAR-T cells have robust and sustained antitumor activity against HER2-positive tumors *in vitro* (A) An impedance-based tumor-cell-killing assay was used to evaluate PB-HER2-CAR-T cells. Tumor cells (U-2OS) were plated, and 24 h later, CAR-T cells were added at different effector-to-target (E:T) ratios. Real-time impedance traces of tumor cells without treatment (green) and with co-culture of CAR-T cells (magenta, blue, and red) were shown. Each experiment was duplicated, and mean ± SD of the normalized cell index at each time point is shown in the graph. (B) Antitumor efficacy of PB-HER2-CAR-T cells against HER2-positive tumor cell lines. Real-time impedance traces of tumor cells without treatment (green) and tumor cells co-cultured with PB-CD19-CAR-T cells (blue) or PB-HER2-CAR-T cells (red) at an E:T ratio of 1:1 were acquired for 72 h. (C) Sequential killing assay of PB-HER2-CAR-T cells. Real-time impedance traces of tumor cells without treatment (green) and tumor cells co-cultured with PB-HER2-CAR-T cells (red) were acquired for 72 h in each round. Mean ± SD of normalized cell index at 72 h in each round were also evaluated (n = 3). (D) The expression of HER2-CAR molecules on T cells during sequential co-culture. Representative dot plots are shown. (E) Mean fluorescent Index of HER2-CAR expression on T cells during sequential co-culture. (F) The expression of PD-1, TIM-3, and LAG3 on HER2-CAR-T cells during sequential co-culture.

We evaluated the ability of PB-HER2-CAR-T cells to kill tumor targets in co-culture assays. PB-HER2-CAR-T cells were co-cultured with the HER2-positive osteosarcoma cell line, U-2OS, at different effector-to-target (E: T) ratios, and the growth of the tumor cells was monitored using the xCELLigence Real Time Cell Analyzer (ACEA Biosciences). Similarly, the antitumor effect of PB-HER2-CAR-T cells on other HER2-positive tumor cell lines, including rhabdomyosarcoma (SJCRH30), Ewing’s sarcoma (RD-ES), synovial sarcoma (SW-982), and invasive breast cancer (BT549 and SK-BR-3) (Figure S9A), were also assessed. PB-HER2-CAR-T cells demonstrated potent and sustained killing activity against U-2OS (Figure 4A) and other HER2-positive tumor cells (Figures 4B and S9B). PB-HER2-CAR-T cells could also eradicate triple-negative breast cancer cells, BT549, which expressed low levels of HER2 and were resistant to HER2 monoclonal antibody-based therapy (Figure S9B).

We also evaluated the persistence of antitumor activity of PB-HER2-CAR-T cells using a tumor re-challenge assay in which fresh tumor cells were added to CAR-T cells every 3 days. PB-HER2-CAR-T cells demonstrated potent and sustained killing activity against U-2OS cells, even after multiple rounds of tumor re-challenges, without deterioration of the antitumor efficacy by T cell exhaustion (Figure 4C and S10), as previously described.^17^ Although it was reported that multiple rounds of antigen stimulation increase CAR expression, leading to tonic CAR signaling and exhaustion,^18^ in our study, the expression of CAR molecule on the cell surface following repeated antigen stimulation did not increase and was controlled at a relatively low level (Figures 4D and 4E). PD-1 expression was gradually, but not significantly, induced during multiple rounds of antigen stimulation (Figure 4F). Strikingly, even the expression of TIM-3 and LAG3 was relatively high at the end of the manufacture, and these expressions gradually decreased during multiple rounds of antigen stimulation (Figure 4F), indicating lower vulnerability to immune exhaustion.

### PB-HER2-CAR-T cells efficiently controlled HER2-positive tumor *in vivo*

**Figure 5:**
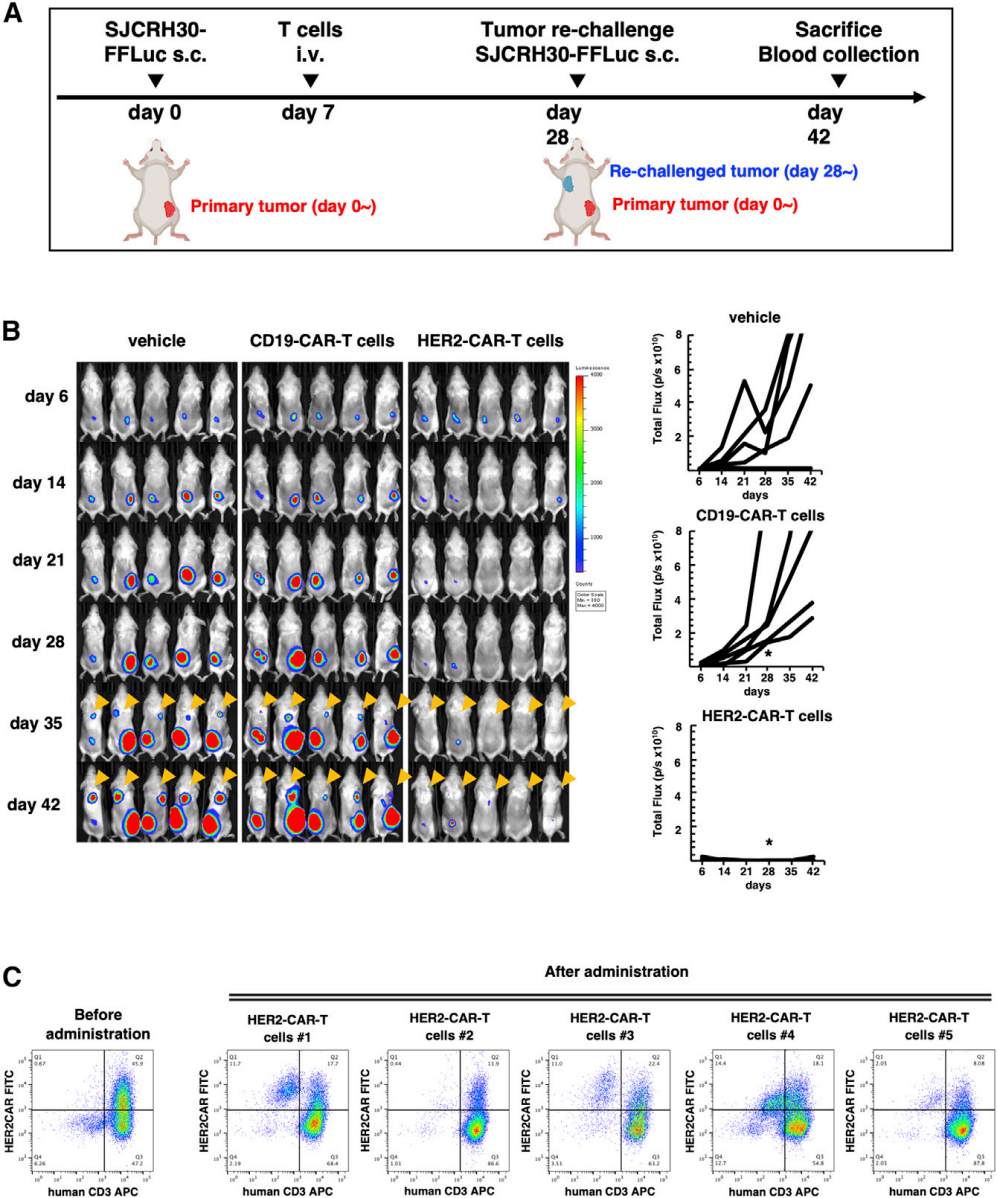
PB-HER2-CAR-T cells efficiently control HER2-positive tumor *in vivo* (A) Schematics of *in vivo* study and tumor re-challenge experiments in the HER2-positive tumor xenograft murine model. SJCRH30-FFluc was subcutaneously injected at the dorsal wall of the mice to form a tumor. One week after tumor engraftment, mice intravenously received either vehicle, approximately 6 × 10^6^ of PB-CD19-CAR-T cells, or PB-HER2-CAR-T cells. (B) Tumor growth was measured as bioluminescence signal intensity (BLI) and expressed as total flux (p/s). The BLI images of each mouse and the tumor volumes measured as total flux (p/s) are shown. The PB-HER2-CAR-T cells group demonstrated a statistically significant tumor reduction measured as the mean total flux at day 28 (∗), compared to the PB-CD19-CAR-T cells group (Mann-Whitney U test, p < 0.01). The SJCRH30-FFLuc tumor cells were re-inoculated into the corresponding contralateral thoracic wall of the mice on day 28 after the primary tumor inoculation. BLI images on days 7 and 14 after tumor re-challenges are indicated by arrowheads. (C) Blood samples were collected from each mouse on day 42 after primary tumor inoculation, and the presence of PB-HER2-CAR-T cells was assessed using flow cytometry. HER2CAR^+^/human CD3^−^ population could be explained as caused by the non-specific binding of anti-human IgG-Fc antibody to mouse PBMCs, as shown in Figure S11.

We established a xenograft mouse model of SJCRH30 cells expressing firefly luciferase (SJCRH30-FFluc) by injecting tumor cells subcutaneously into non-obese diabetic severe combined immunodeficiency (interleukin-2) IL-2 receptor γ-chain-deficient (NSG) mice. After establishing the tumor mass 7 days after inoculation, vehicle, PB-CD19-CAR-T cells, or PB-HER2-CAR-T cells were intravenously injected, and the antitumor efficacy of CAR-T cells was evaluated by monitoring tumor growth using *in vivo* luminescence (Figure 5A). Mice that received vehicle or PB-CD19-CAR-T cells developed huge tumor masses and reached humane endpoints around day 42. By contrast, the tumor treated with PB-HER2-CAR-T cells were effectively controlled and even disappeared in some mice, indicating the antitumor efficacy of PB-HER2-CAR-T cells *in vivo* (Figure 5B).

To further investigate the protective immune memory response against tumor establishment, we re-inoculated tumor cells at the contralateral site of the surviving mice on day 28 following initial treatment. Mice treated with vehicle or PB-CD19-CAR-T cells developed tumor masses 7 days after tumor re-inoculation, whereas mice treated with PB-HER2-CAR-T cells were resistant to second tumor engraftment (Figure 5B, arrowhead).

The fact that PB-HER2-CAR-T cells exhibited a memory-rich phenotype even after the expansion (Figure 3C) prompted us to hypothesize that the stable antitumor activity of PB-HER2-CAR-T cells would be attributable to the prolonged persistence of PB-HER2-CAR-T cells *in vivo*. To explore the *in vivo* persistence of PB-HER2-CAR-T cells in tumor-bearing mice, we collected a blood sample from mice treated with PB-HER2-CAR-T cells on day 42 after the initial treatment and assessed the presence of PB-HER2-CAR-T cells using flow cytometry. As anticipated, PB-HER2-CAR-T cells were detected in abundance in mice that showed tumor rejection (Figure 5C), indicating their prolonged existence *in vivo*, while maintaining their killing capacity even after tumor eradication.

## Discussion

PB-based gene transfer was introduced as a faster, safer, and cost-effective system for T cell engineering compared to other types of viral gene transfer,^19^ and attempts have been made to manufacture clinical-grade PB-CAR-T cells and to overcome the relatively low transduction efficacy. It has been shown that the activation-induced cell death caused by excess stimulation of T cells by antibodies impairs cell expansion of electroporated T cells;^13^ thus, researchers have been using feeder cells for optimized stimulation of T cells. Previous studies have also demonstrated that the use of tumor-cell-based feeder cells or PBMCs enhance the efficacy of manufacturing transposon mediated CAR-T cells.^13^^,^^15^^,^^20^ K562-based feeder cells could be applied for the stimulation of SB transposon-mediated CAR-T cells to overcome the low transduction efficiency; however, the stimulation of tumor cell-based feeder cells requires a relatively longer expansion period and induces the enrichment of effector and exhausted T cells.^9^^,^^21^ These data imply that tumor-cell-based feeder cells should be further optimized to enrich memory-like CAR-T cells that determine the function of CAR-T cells.^2^ PBMCs, another T cell activator, have also been used for the expansion of non-virally engineered CAR-T cells. Ramanayake et al.^20^ reported that PB-CD19-CAR-T cells exhibited > 75% CAR positivity and > 100-fold expansion during 21 days of *ex vivo* culture with three rounds of stimulation with PBMCs every 7 days. However, these CAR-T cells require multiple rounds of stimulation with PBMCs, which might cause enrichment of effector cells.^20^ Even though we utilized known manufacturing procedures for PB-CD19-CAR-T cells^13^^,^^15^^,^^20^ to produce PB-HER2-CAR-T cells, we could not obtain HER2-CAR-positive T cells (data not shown). The precise mechanism of the beneficial effect of PBMCs for manufacturing PB-CD19-CAR-T cells remains to be elucidated. Nonetheless, we speculate that CD19-CAR-T cells may be stimulated by CD19-expressing B cells in PBMCs via the CAR molecule, which may mimic the physiological T cell receptor/major histocompatibility complex (TCR/MHC) interaction and support the expansion of CAR-T cells. Moreover, co-stimulatory molecules expressed on B cells could complement the interaction of the CD19/CD19CAR molecule, leading to complete activation and expansion of CAR-T cells. Hence, the antigen-presenting feeder cells could provide physiological interaction mimicking the immune synapses through the CAR molecule and ample co-stimulation, leading to the optimal activation and expansion of memory-rich CAR-T cells.

To overcome the limited effects of CAR-T therapy for solid tumors, identifying and overcoming the mechanisms associated with dysfunction of CAR-T cells is important to increase its efficacy. The quality of CAR-T cell products characterized by memory-rich phenotype and low expression of exhaustion marker is important to improve the efficacy of CAR-T cell therapy.^2^ The mechanisms underlying exhaustion are multifactorial and remain poorly understood. One possible cause of PB-CAR-T cells maintaining the undifferentiated phenotype without the induction of PD-1 during expansion in our study may be co-existence of ample co-stimulatory molecules together with antigens in *cis* on the antigen-presenting feeder cells. Recent studies illustrated that when antigen-presenting cells express substantial amounts of CD80, this molecule can interact with PD-L1 in *cis* to disrupt PD-L1/PD-1 binding, thereby inhibiting the activation of PD-1 and positive feedback of PD-1 overexpression on T cells.^22^^,^^23^ These reports support our observation that the *cis*-expressed CD80 together with truncated HER2 on antigen-presenting cells could limit the PD-1 co-inhibitory signal on T cells, while promoting CD28-mediated co-stimulation, identifying critical components for induction of optimal T cell stimulation.^22^^,^^23^

A limitation of PB-CAR-T cells is the relatively lower expansion of CAR-T cells than virally engineered CAR-T cells previously reported, which might be attributable to electroporation-induced cell death.^24^ Moreover, the electrotransfer of a large DNA plasmid results in low viability due to DNA toxicity.^25^ Therefore, the electroporation protocol and the transgene vector should also be optimized to reduce cellular damage by electroporation or DNA toxicity. The use of minicircle vector or mRNA instead of DNA plasmid to introduce PB transposase may also improve viability and transduction efficacy.^10^^,^^26^ Moreover, the optimization of manufacturing procedures should also be performed using heavily pre-treated patients’ samples before the clinical trial, since the manufacturing failure could occur in the samples from pre-treated patients.^27^^,^^28^

In conclusion, we developed a manufacturing procedure for PB-HER2-CAR-T cells. Our expansion procedure involved autologous PBMC-based antigen-presenting cells expressing ample co-stimulatory molecules and demonstrated high transduction efficiency of HER2-CAR transgene and T cell expansion. PB-HER2-CAR-T cells demonstrated a memory-rich phenotype with minimal PD-1 expression, leading to sustained tumor control *in vitro* and *in vivo*. Our manufacturing process has the potential for producing various CAR-T cells enriched with the memory phenotype associated with stable antitumor efficacy and scalability for clinical use. This can be achieved without a notoriously expensive viral vector production core and regulation step to validate the contamination of replication-competent viruses.

## Materials and methods

### Ethics approval and consent to participate

This study was approved by the Institutional Review Board of Shinshu University (approval no. 19-011) and Kyoto Prefectural University of Medicine (approval no. 2019-111 and 2019-112). All blood samples from healthy donors were obtained with written informed consent using the protocol approved by the Institutional Review Board of Shinshu University School of Medicine (approval no. 4265) and Kyoto Prefectural University of Medicine (approval no. ERB-C-669 and ERB-C-1406). All animal experiments were performed with protocols approved by the Shinshu University School of Medicine Institutional Animal Care and Use Committee (approval no. 019102).

### Cell line and human blood samples

The HER2-positive tumor cell lines U-2OS (HTB-96), SJCRH30 (CRL-2061), RD-ES (HTB-166), SW-982 (HTB-93), and BT549 (HTB-122) were purchased from the American Type Culture Collection. SK-BR-3 cells were kindly provided from Dr. Ken-ichi Ito (Shinshu University). SJCRH30 expressing FFluc (SJCRH30-FFluc) was obtained by introducing PB-based pIRII-FFLuc-puroR-GFP, which encodes the FFLuc gene, puromycin-resistant gene, and green fluorescent protein gene, on SJCRH30 cells by electroporation, and subsequent cloning to obtain single-cell-derived clone. SJCRH30 and SJCRH30-FFLuc cells were cultured in Dulbecco’s modified Eagle’s medium (Thermo Fisher Scientific), supplemented with 10% fetal bovine serum (FBS) (Cytiva) and 1% penicillin-streptomycin (Thermo Fisher Scientific). U-2OS and SK-BR-3 cells were cultured in McCoy’s 5A medium (Thermo Fisher Scientific), supplemented with 10% FBS and 1% penicillin-streptomycin. RD-ES and BT549 cells were cultured in RPMI-1640 medium (Gibco), supplemented with 10% FBS (Cytiva) and 1% penicillin-streptomycin (Thermo Fisher Scientific). SW-982 cells were cultured in DMEM/F12 (Thermo Fisher Scientific), supplemented with 10% FBS (Cytiva) and 1% penicillin-streptomycin (Thermo Fisher Scientific). All cells were maintained in a humidified incubator at 37°C in a 5% CO_2_ atmosphere. All cells were passaged for less than 6 months before use and were periodically authenticated by morphologic inspection and tested for mycoplasma. Blood samples from healthy donors were obtained in ethylenediaminetetraacetic acid-sodium (EDTA-2Na) tubes with written informed consents using the protocol approved by the Institutional Review Board of Shinshu University and Kyoto Prefectural University of Medicine and immediately used for CAR-T cell manufacture.

### Plasmid construction

pCMV-PB plasmid encoding PB transposase that was described previously^4^ was artificially synthesized (Mediridge, Tokyo, Japan). CAR construct encoding the HER2-specific single-chain variable fragment (scFv), followed by a short hinge, the transmembrane and signaling domain of the costimulatory molecule CD28, and the ζ-signaling domain of the TCR, was kindly provided by Dr. Stephen Gottschalk (St. Jude Children’s Research Hospital) and was subcloned into pIRII transposon vector backbone (pIRII-HER2-28z) as described previously^5^ (Figure S1). The CAR construct for CD19-CAR-T cells, which encodes the CD19-specific scFv, followed by a short hinge, the transmembrane and signaling domain of the costimulatory molecule CD28, and the ζ-signaling domain of the TCR complex, was kindly provided from Dr. Cliona M. Rooney (Baylor College of Medicine) and was subcloned into pIRII transposon vector backbone as described previously.^6^ GD2-CAR, which encodes the GD2-specific scFv, followed by a short hinge, immunoglobulin G (IgG)-Fragment Crystallizable region (Fc) spacer, the transmembrane and signaling domain of the costimulatory molecule CD28 or 4-1BB, and the ζ-signaling domain of the TCR complex,^29^^,^^30^ was kindly provided from Dr. Cliona M. Rooney (Baylor College of Medicine) and was subcloned into pIRII transposon vector backbone (pIRII-GD2-28z and pIRII-GD2-BBz, respectively) (Figure S1). For the generation of antigen-presenting feeder cells for the stimulation of HER2-CAR-transduced T cells, we used a plasmid containing sequences encoding the extracellular, transmembrane, and 50 amino-acid-long intracellular portion of HER2 protein (tHER2) driven by CMV promoter, followed by CD80 and 4-1BBL (CD137L) with P2A self-cleaving sites driven by EF1α promoter that enabled independent gene expression. The tHER2-CD80-4-1BBL sequence was artificially synthesized (Fasmac, Kanagawa, Japan) and cloned into a pIRII PB transposon vector (pIRII-tHER2-CD80-4-1BBL) (Figure S1). A plasmid for antigen-presenting feeder cells for the stimulation of CD19-CAR-T cells was created by replacing the tHER2-CD80-4-1BBL portion of pIRII-tHER2-CD80-4-1BBL with truncated CD19 sequences encoding the extracellular, transmembrane, and 20-amino-acid intracellular portion of CD19 protein, CD80, and 4-1BBL with T2A and P2A self-cleaving sites (Figure S1). Similarly, a plasmid for antigen-presenting feeder cells for the stimulation of GD2-CAR-T cells was created by replacing the tCD19 portion of pIRII-tCD19-CD80-4-1BBL with the sequence of GD2 synthase (B4GALNT1) and GD3 synthase (ST8SIA1) (Figure S1).

### Manufacturing PB-mediated CAR-T cells

PBMCs were isolated from whole-blood samples by density gradient centrifugation using lymphocyte separation medium 1077 (Fujifilm Wako Pure Chemical Corporation, Osaka, Japan), followed by multiple washes in phosphate-buffered saline (PBS; Fujifilm Wako Pure Chemical Corporation). The number of live cells was determined using standard trypan-blue staining and automated cell counter model R1 (Olympus, Tokyo, Japan). On day 0, 20 × 10^6^ fresh PBMCs were electroporated with 7.5 μg pCMV-PB plasmid and 5 μg CAR transgene plasmids (pIRII-HER2-28z, pIRII-CD19-28z, pIRII-GD2-28z, and pIRII-GD2-BBz) for HER2-, CD19-, and GD2-CAR-T cells, respectively. Concurrently, 20 × 10^6^ fresh PBMCs from the same donor were also electroporated with 12.5 μg antigen-presenting feeder plasmid (pIRII-tHER2-CD80-4-1BBL, pIRII-tCD19-CD80-4-1BBL, and pIRII-B4GALNT1/ST8SIA1-CD80-4-1BBL) for antigen-presenting feeder cells on day 0. Electroporation was performed using the P3 Primary Cell 4D-Nucleofector × kit (Lonza, Basel, Switzerland, Program; FI-115) or MaxCyte ATX (MaxCyte, Gaithersburg, MD, USA) with the optimized protocol for the introduction of DNA plasmid into resting T cells (Protocol; RTC 14-3). After electroporation, CAR-T and antigen-presenting feeder cells were cultured in CCM consisting of ALyS 705 medium (Cell Science & Technology Institute, Miyagi, Japan) supplemented with 5% artificial serum (animal-free; Cell Science & Technology Institute), IL-7 (10 ng/mL, corresponding to an activity of 500 U/mL, Miltenyi Biotec, Bergisch Gladbach, Germany), and IL-15 (5 ng/mL corresponding to an activity of 25 U/mL, Miltenyi Biotec), in a 37°C, 5% CO_2_ atmosphere humidified incubator for 24 h. On day 1, the feeder cells were inactivated by ultraviolet (UV) irradiation and subsequently co-cultured with CAR-T cells in CCM for 14 days. Half of the culture supernatant was replaced with fresh medium every 2 days (Figure 3A).

### Optimization analysis of T cell expansion

Cell proliferation was determined by cell counting using the automated cell counter model R1 (Olympus). For the stimulation of T cells by plate-bound anti-CD3 and -CD28 antibodies, 24-well not-treated multiple well plates (Corning, Corning, NY, USA) were treated with 1 μg/mL of anti-CD3 and -CD28 monoclonal antibodies (BioLegend, San Diego, CA, USA) overnight at 4°C, and then HER2-CAR-transduced T cells were cultured with CCM supplemented with IL-7 and IL-15 as described above, on an anti-CD3 and -CD28 monoclonal antibody-coated plate for 48 h. For the stimulation of T cells by tumor cells, 3 × 10^6^ SJCRH30 cells were UV-irradiated for inactivation and cultured with 3 × 10^6^ HER2-CAR transduced T cells for 48 h.

### Flow cytometry

Cell-surface expression of the HER2-CAR molecules on PB-HER2-CAR-T cells or CD19-CAR molecules on PB-CD19-CAR-T cells was determined using the flow cytometry using recombinant human ErbB2/HER2-Fc chimera protein or recombinant human CD19 Fc chimera protein, respectively (R&D Systems, Minneapolis, MN, USA), followed by a goat anti-human IgG Fc fragment-specific antibody conjugated to fluorescein isothiocyanate (FITC; Merck Millipore, Burlington, MA, USA). Cell-surface expression of the GD2-CAR molecule on PB-GD2-CAR-T cells was determined using flow cytometry using a goat anti-human IgG Fc fragment-specific antibody conjugated to FITC (Merck Millipore). Phycoerythrin (PE) or allophycocyanin-conjugated antibodies CD3, CD4, CD8, CD45RA, CCR7, and PD-1 (BioLegend, San Diego, CA, USA) were used for the characterization of the phenotype of CAR-T cells. Detailed information of recombinant protein and antibody used was shown in Table S1. All flow cytometry data was acquired using BD Accuri C6 Plus or BD FACSLyric (BD Biosciences) and analyzed using the FlowJo Software (BD Biosciences).

### Cytotoxicity assay

HER2-positive tumor cells were plated on xCELLigence E-plates 16 (ACEA Biosciences, San Diego, CA, USA) at a density of 0.5–2 × 10^4^/well 18–24 h prior to CAR-T cell seeding. HER2-CAR-T cells or CD19-CAR-T cells were added at an E:T ratio of 1:4, 1:2, or 1:1; then real-time impedance was measured for 72 h and presented as the normalized cell index using an xCELLigence RTCA DP system. Data were analyzed using Software v2.0 (ACEA Biosciences).

### Sequential killing assay

We seeded 1 × 10^4^ U-2OS cells on E-plate 16 (ACEA Biosciences) 24 h before co-culture, and then added PB-HER2-CAR-T cells at an E:T ratio of 1:1. The killing effect of the PB-HER2-CAR-T cells was analyzed using xCELLigence RTCA DP system (ACEA Biosciences). Concurrently, we seeded 1 × 10^5^ U-2OS cells on a 24-well plate 24 h before co-culture and added PB-HER2-CAR-T cells at an E:T ratio of 1:1. Three days later, the CAR-T cells were collected from the 24-well plate and counted, and then 1 × 10^4^ CAR-T cells were co-cultured with 1 × 10^4^ fresh U-2OS cells on E-plate 16, which was prepared 24 h before second co-culture. Cell counting and re-plating were repeated every 3 days with 3 iterations.

### *In vivo* xenograft HER2-positive sarcoma model

Female 6-week-old NSG mice were purchased from Charles River Laboratories Japan and housed at the Shinshu University School of Medicine for over a week before the experiment. Food and water were available ad libitum. SJCRH30-FFluc cells were suspended in a total volume 100 μL of PBS to prepare 1 × 10^6^ cells and subcutaneously injected to the dorsal wall of the mice to form a tumor. One week after tumor inoculation, either vehicle, approximately 6 × 10^6^ of PB-CD19-CAR-T cells or PB-HER2-CAR-T cells, were intravenously administered to the mice via tail vein. Tumor engraftment was measured as bioluminescence signal intensity (BLI) and expressed as total flux (p/s) using the *in vivo* imaging system (IVIS Lumina LT, PerkinElmer, Waltham, MA, USA). All the images were taken 10 min after intraperitoneal injection of luciferin (Promega, Madison, WI, USA) at 0.3 mg/mouse, with 1 s acquisition and 4 of binning. Image analysis and bioluminescent quantification were performed using Living Image Software (PerkinElmer). For tumor re-challenge experiments, 1 × 10^6^ SJCRH30-FFluc cells were injected into the corresponding contralateral thoracic wall of the mice on day 28. All procedures were performed in a sterile atmosphere and with the mice being anesthetized using 2% isoflurane (Pfizer, New York, NY, USA). To observe the existence of infused CAR-T cells, mice were humanely euthanized by cervical dislocation under anesthesia by trained animal care staff on day 42, and blood samples were collected for further flow cytometry analysis.

### Statistics

Data are presented as means ± standard deviation (SD), unless otherwise stated. Statistical analysis was also performed using GraphPad Prism 7 software (GraphPad Software, San Diego, CA, USA) to perform two-sided t test or Mann-Whitney U test. Probability values of p <0.05 were considered significant.

## Supplemental information

Document S1. Figures S1–S11 and Table S1

Document S2. Article plus supplemental information
