# Supplementary material for: Autologous antigen-presenting cells efficiently expand piggyBac transposon CAR-T cells with predominant memory phenotype
Source: Mol Ther Methods Clin Dev. 2021 Mar 23;21:315–24. doi: 10.1016/j.omtm.2021.03.011 (PMC8047430; doi:10.1016/j.omtm.2021.03.011)
Supplement: Document S1. Figures S1–S11 and Table S1 [file mmc1.pdf]

**OMTM, Volume *21***

## Supplemental information

# Autologous antigen-presenting cells efficiently expand *piggyBac* transposon CAR-T cells with predominant memory phenotype

**Kayoko Nakamura, Shigeki Yagyu, Shogo Hirota, Akimasa Tomida, Makoto Kondo, Tomokuni Shigeura, Aiko Hasegawa, Miyuki Tanaka, and Yozo Nakazawa**

## Supplemental Information

### Figure S1

Transposon plasmids for CAR-T cells and antigen-presenting feeder cells. ITR; Internal Tandem Repeat, TM; transmembrane domain, cyto; cytoplasmic domain.

### Figure S2

HER2-CAR expression 24 h after electroporation. Representative dot plot Data from 3 different donors are shown.

### Figure S3

Expression of CD80, 4-1BBL, CD86 and OX40 on unmanipulated T cells. Data from 3 different donors are shown.

### Figure S4

Composition of antigen-presenting feeder cells. **a.** Percentage of truncated HER2-positive/CD3-, CD14-, CD19, or CD56-pototive cells in feeder cells 24 h post-electroporation. Dot plot data from 3 different donors are shown. b. Mean ± SD percentage of HER2+/CD3+, HER2+/CD14+, HER2+/CD19+, and HER2+/CD56+ cells in feeder cells (n=3).

### Figure S5

Detailed phenotype analysis of PB-HER2-CAR-T cells **a.** Expression of TIM-3 and LAG3 on PB-HER2-CAR-T cells established from three different donors. **b.** Expression of CD28/CD95 in CD45RA/CCR7 double positive fraction and **c.** differentiation profiles in CD4-positive or CD8-positive subpopulations of PB-HER2-CAR-T cells established from three different donors.

### Figure S6

PB-HER2-CAR-T cells expanded by PBMC-derived feeder cells expressing truncated HER2. **a.** plasmid construct for feeder cells expressing HER2 (pIRII-tHER2). We introduced pIRII-tHER2 or pIRII-tHER2-CD80-4-1BBL, into PBMC to produce antigen presenting feeder cells, and generate PB-HER2-CAR-T cells, respectively, according to the same manufacturing procedure described in Materials and Methods. **b.** Representative dot plot shows the phenotype of PB-HER2-CAR-T cells expanded by HER2-expressing feeder cells or HER2, CD80, 4-1BBL-expressing feeder cells. The population of CD45RA+/CCR7+ T stem cell memory-like cells in CAR+ cells were 19.6% (HER2 expressing feeder cells) and 66.5% (HER2, CD80, and 4-1BBL expressing feeder cells), respectively. **c.** Antitumor efficacy of PB-HER2-CAR-T cells stimulated by feeder cells expressing HER2 (HER2 expressing feeder cells) or HER2, CD80, and 4-1BBL (HER2, CD80, and 4-1BBL expressing feeder cells) against SJCRH30 cells. Real-time impedance traces of tumor cells with (red and blue) or without treatment (green) at an E:T ratio of 1:1 were acquired for 72 hours.

**Figure S7**

CAR expression and phenotype of PB-CD19-CAR-T cells. pIRII-CD19-28z (Figure S1) were introduced into resting T cells on day 0, then co-cultured with CAR stimulating feeder cells expressing truncated CD19 together with CD80 and 4-1BBL, and cultured as described in Materials and Methods.

**Figure S8**

PB-GD2-CAR-T cells manufactured by CAR stimulating feeder cells. **a.** CAR expression and phenotype of PB-GD2-28z-CAR-T cells and **b.** GD2-BBz-CAR-T cells. GD2-28z, GD2-BBz transgene (Figure S1) were introduced into resting T cells on day 0, then co-cultured with CAR stimulating feeder cells expressing GD2/GD3 synthase together with CD80 and 4-1BBL, and cultured as described in Materials and Methods.

**Figure S9**

**a.** Expression of HER2 on various tumor cell lines determined by flow cytometry. **b.** Antitumor efficacy of PB-HER2-CAR-T cells against HER2-amplified (SK-BR-3) or triple negative (BT549) breast cancer cell lines. Real-time impedance traces of tumor cells without treatment (green) and tumor cells co-cultured with PB-CD19-CAR-T cells (blue) or PB-HER2-CAR-T cells (red) at an E:T ratio of 1:1 were acquired for 72 hours.

**Figure S10**

Sequential killing assay of PB-HER2-CAR-T cells from donor 2 and 3. Real-time impedance traces of tumor cells (green) and tumor cells co-cultured with PB-HER2-CAR-T cells (red) were acquired for 72 hours in each round.

**Figure S11**

Non-specific binding of anti-human IgG-Fc antibody to mouse PBMC. PBMCs were isolated from untreated mice, then the PBMCs were stained with HER2-Fc chimera protein, anti-human IgG-Fc antibody, and anti-human CD3 antibody as described in Material and Methods. Mice PBMC (human CD3 negative population) were stained with anti-human IgG-Fc.

**Supplemental Table 1**

List of antibodies used in this study

# Supplemental Figure S1

Corresponding Author; Shigeki Yagyu

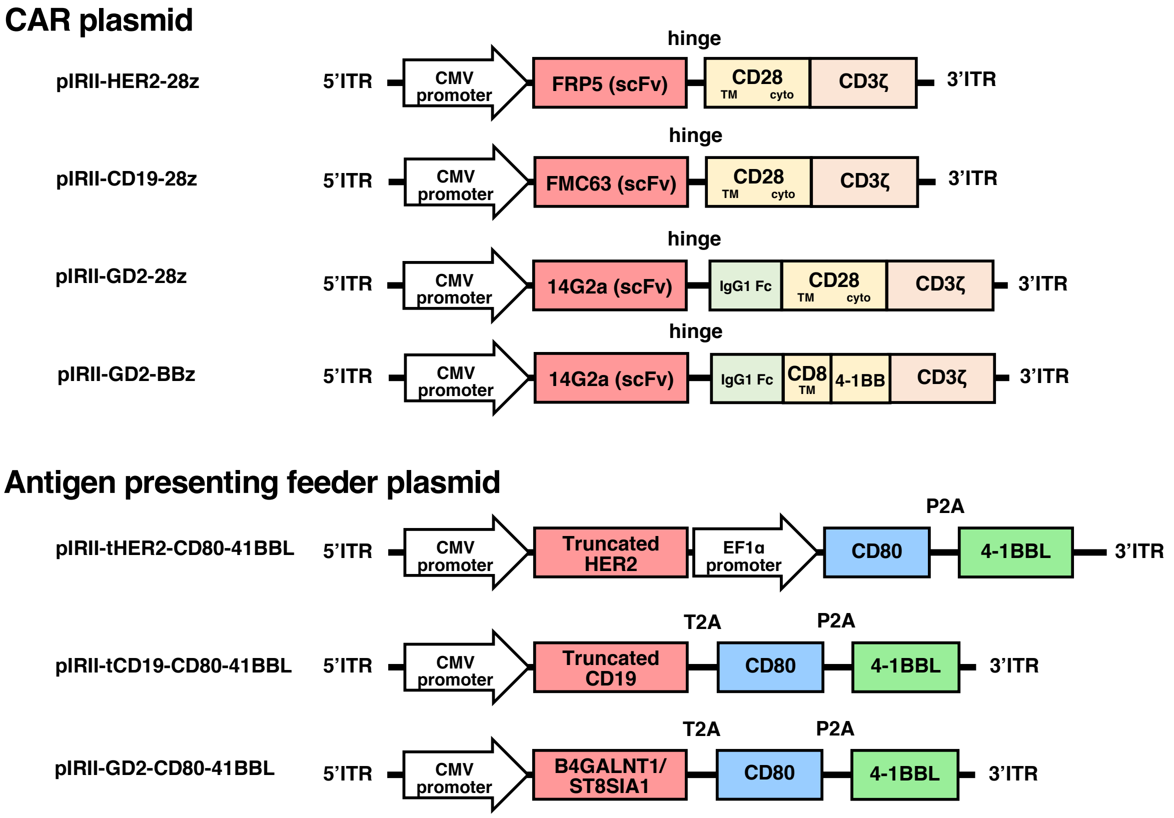


**Figure S1**
Transposon plasmids for CAR-T cells and antigen-presenting feeder cells. ITR; Internal Tandem Repeat, TM; transmembrane domain, cyto; cytoplasmic domain.

# Supplemental Figure S2

Corresponding Author; Shigeki Yagyu

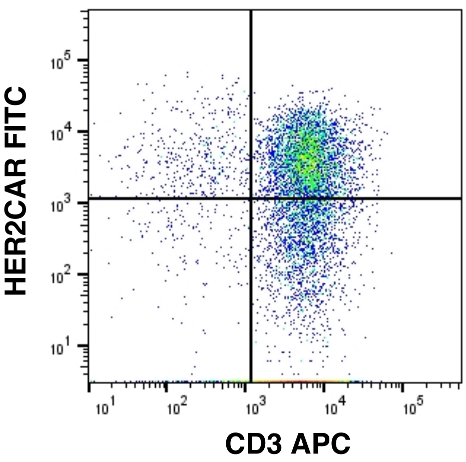


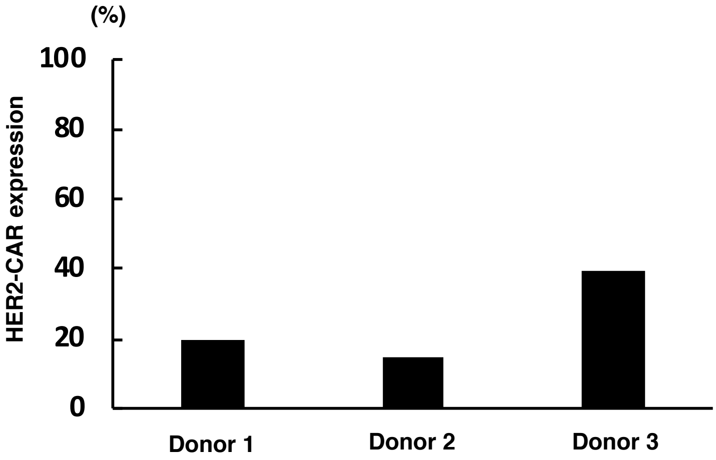


**Figure S2**
HER2-CAR expression 24 h after electroporation. Representative dot plot Data from 3 different donors are shown.

# Supplemental Figure S3

Corresponding Author; Shigeki Yagyu

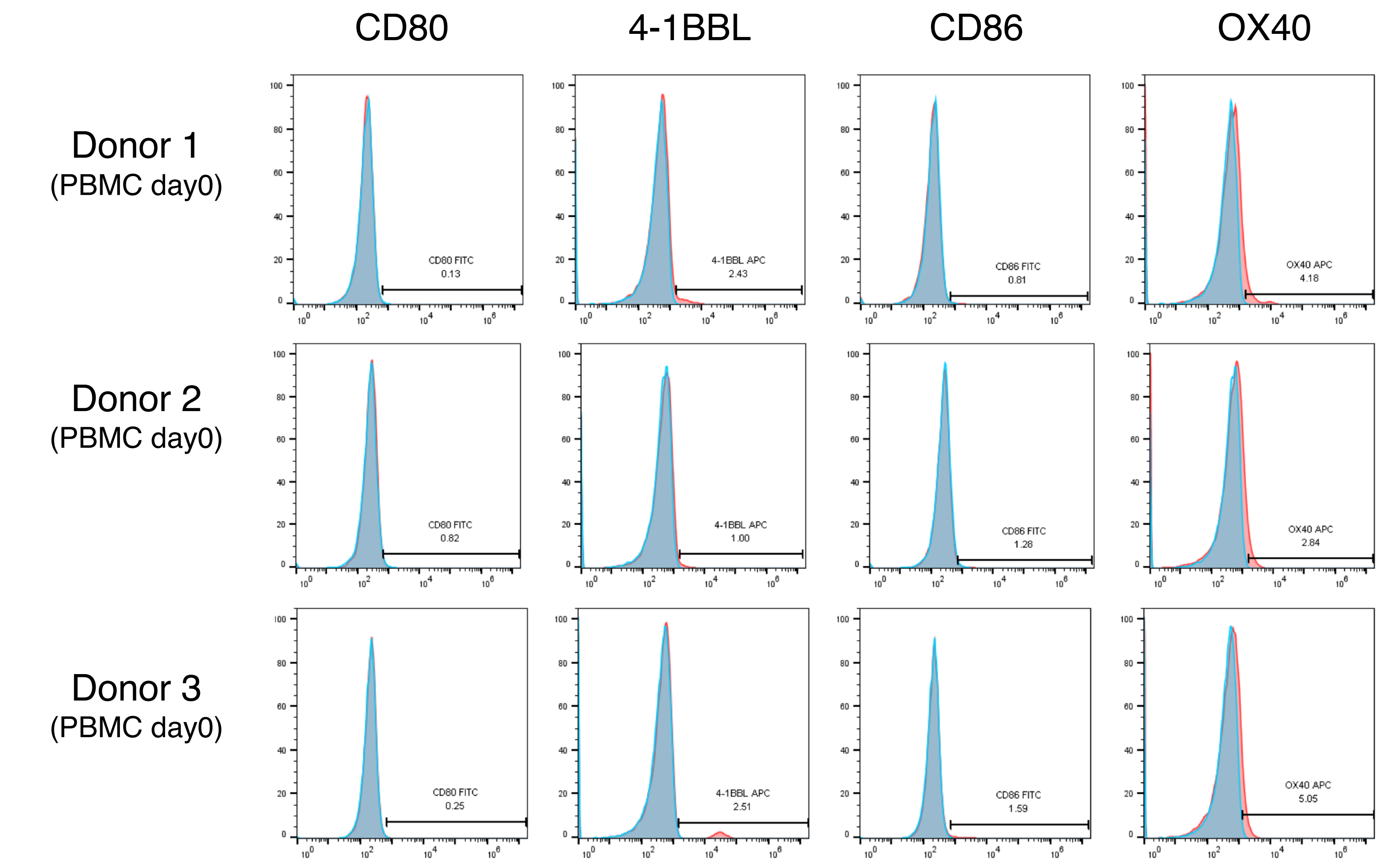


**Figure S3**
Expression of CD80, 4-1BBL, CD86 and OX40 on unmanipulated T cells. Data from 3 different donors are shown.

# Supplemental Figure S4

Corresponding Author; Shigeki Yagyu

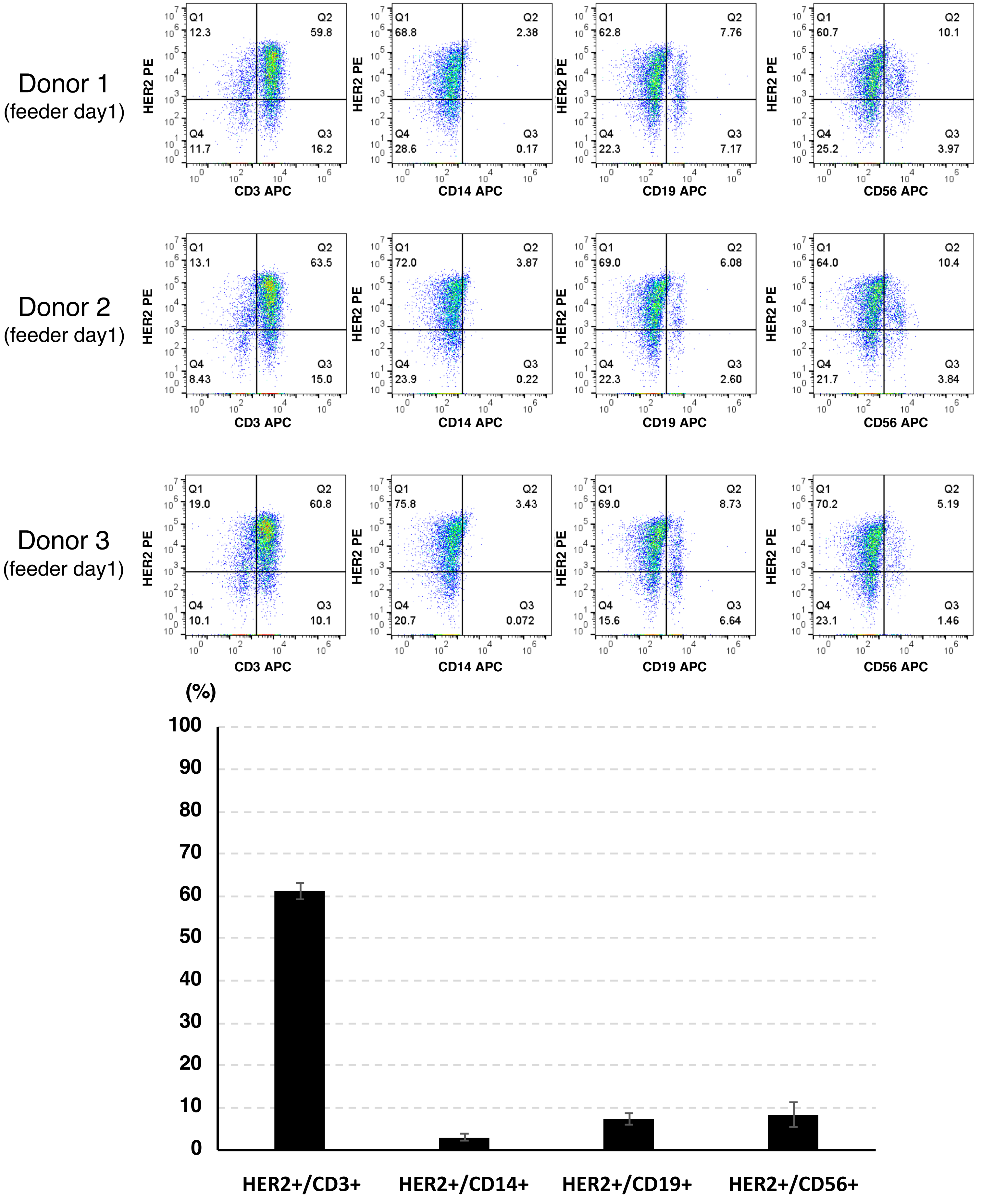


**Figure S4**

Composition of antigen-presenting feeder cells. a. Percentage of truncated HER2-positive/CD3-, CD14-, CD19, or CD56-pototive cells in feeder cells 24 h post-electroporation. Dot plot data from 3 different donors are shown. b. Mean ± SD percentage of HER2+/CD3+, HER2+/CD14+, HER2+/CD19+, and HER2+/CD56+ cells in feeder cells (n=3).

Supplemental Figure S5

Corresponding Author; Shigeki Yagyu

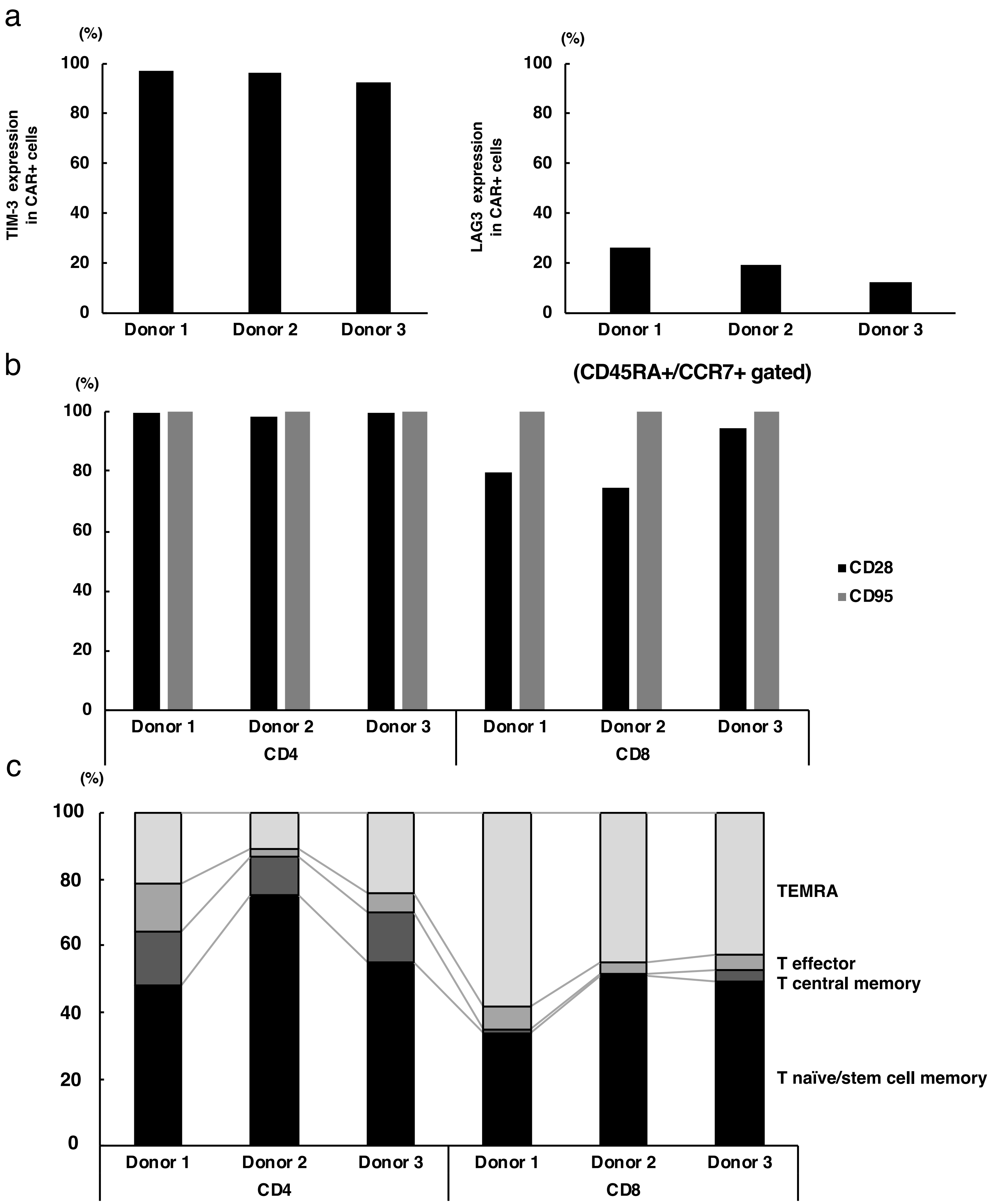


**Figure S5**
Detailed phenotype analysis of PB-HER2-CAR-T cells **a.** Expression of TIM-3 and LAG3 on PB-HER2-CAR-T cells established from three different donors. **b.** Expression of CD28/CD95 in CD45RA/CCR7 double positive fraction and **c.** differentiation profiles in CD4-positive or CD8-positive subpopulations of PB-HER2-CAR-T cells established from three different donors.

# Supplemental Figure S6

Corresponding Author; Shigeki Yagyu

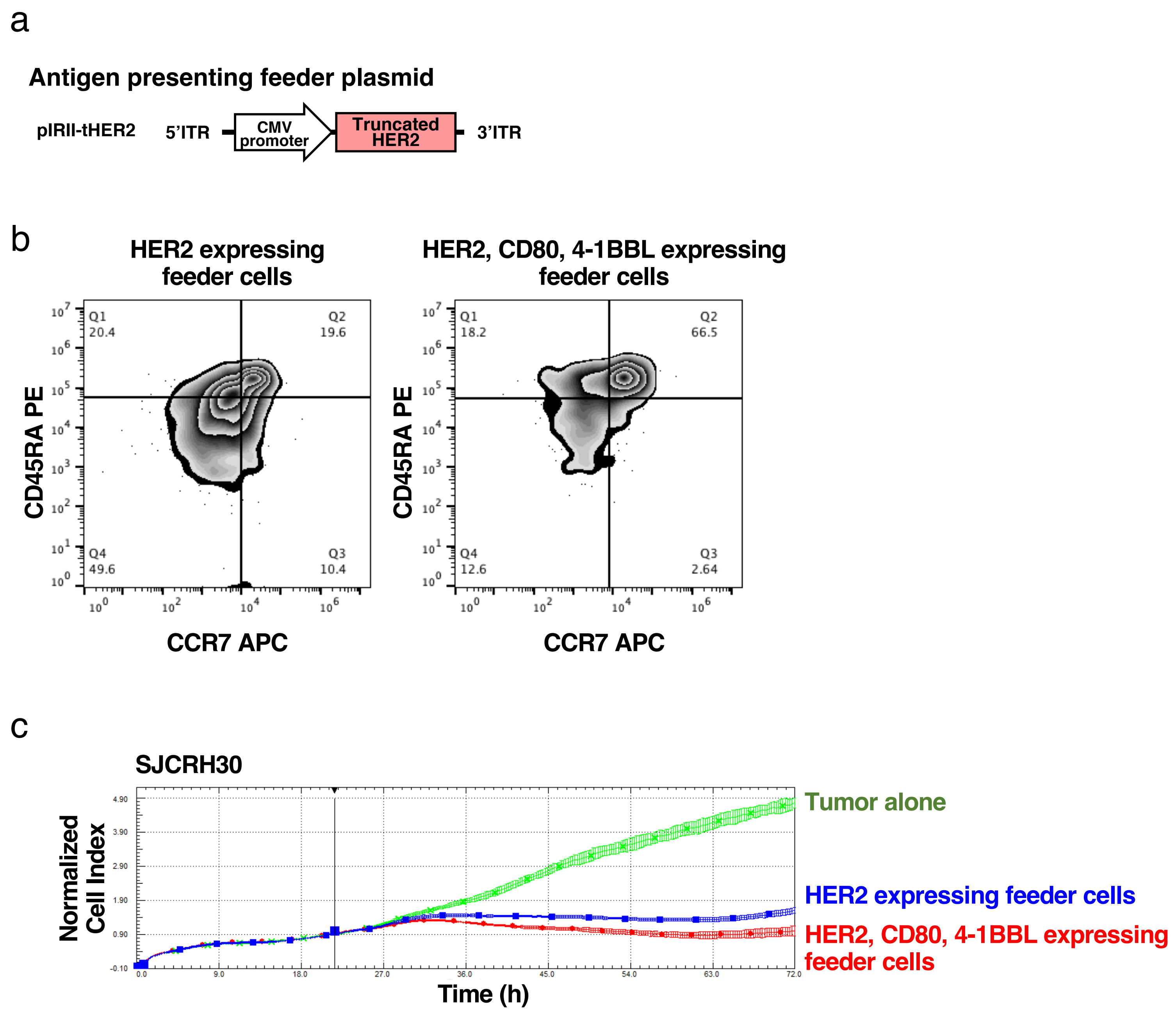


**Figure S6**

PB-HER2-CAR-T cells expanded by PBMC-derived feeder cells expressing truncated HER2. **a.** plasmid construct for feeder cells expressing HER2 (pIRII-tHER2). We introduced pIRII-tHER2 or pIRII-tHER2-CD80-4-1BBL, into PBMC to produce antigen presenting feeder cells, and generate PB-HER2-CAR-T cells, respectively, according to the same manufacturing procedure described in Materials and Methods. **b.** Representative dot plot shows the phenotype of PB-HER2-CAR-T cells expanded by HER2-expressing feeder cells or HER2, CD80, 4-1BBL-expressing feeder cells. The population of CD45RA+/CCR7+ T stem cell memory-like cells in CAR+ cells were 19.6% (HER2 expressing feeder cells) and 66.5% (HER2, CD80, and 4-1BBL expressing feeder cells), respectively. **c.** Antitumor efficacy of PB-HER2-CAR-T cells stimulated by feeder cells expressing HER2 (HER2 expressing feeder cells) or HER2, CD80, and 4-1BBL (HER2, CD80, and 4-1BBL expressing feeder cells) against SJCRH30 cells. Real-time impedance traces of tumor cells with (red and blue) or without treatment (green) at an E:T ratio of 1:1 were acquired for 72 hours.

 Corresponding Author; Shigeki Yagyu

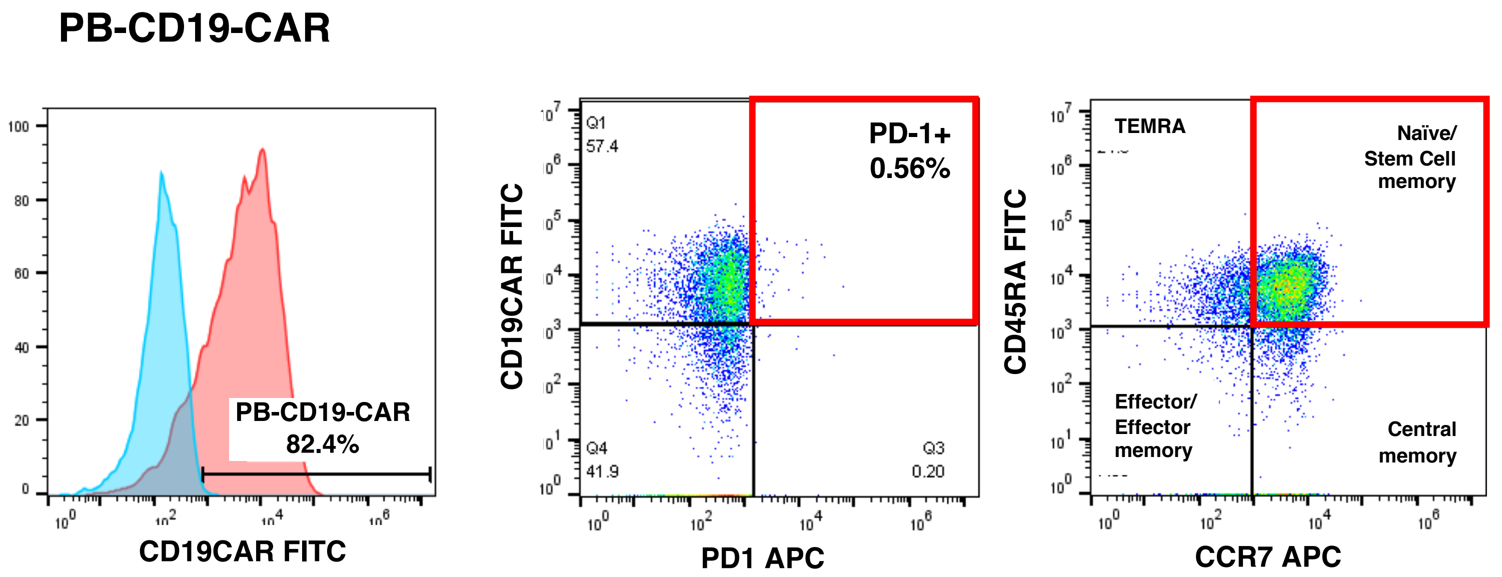


**Figure S7**
CAR expression and phenotype of PB-CD19-CAR-T cells. pIRII-CD19-28z (Figure S1) were introduced into resting T cells on day 0, then co-cultured with CAR stimulating feeder cells expressing truncated CD19 together with CD80 and 4-1BBL, and cultured as described in Materials and Methods.

# Supplemental Figure S8

Corresponding Author; Shigeki Yagyu

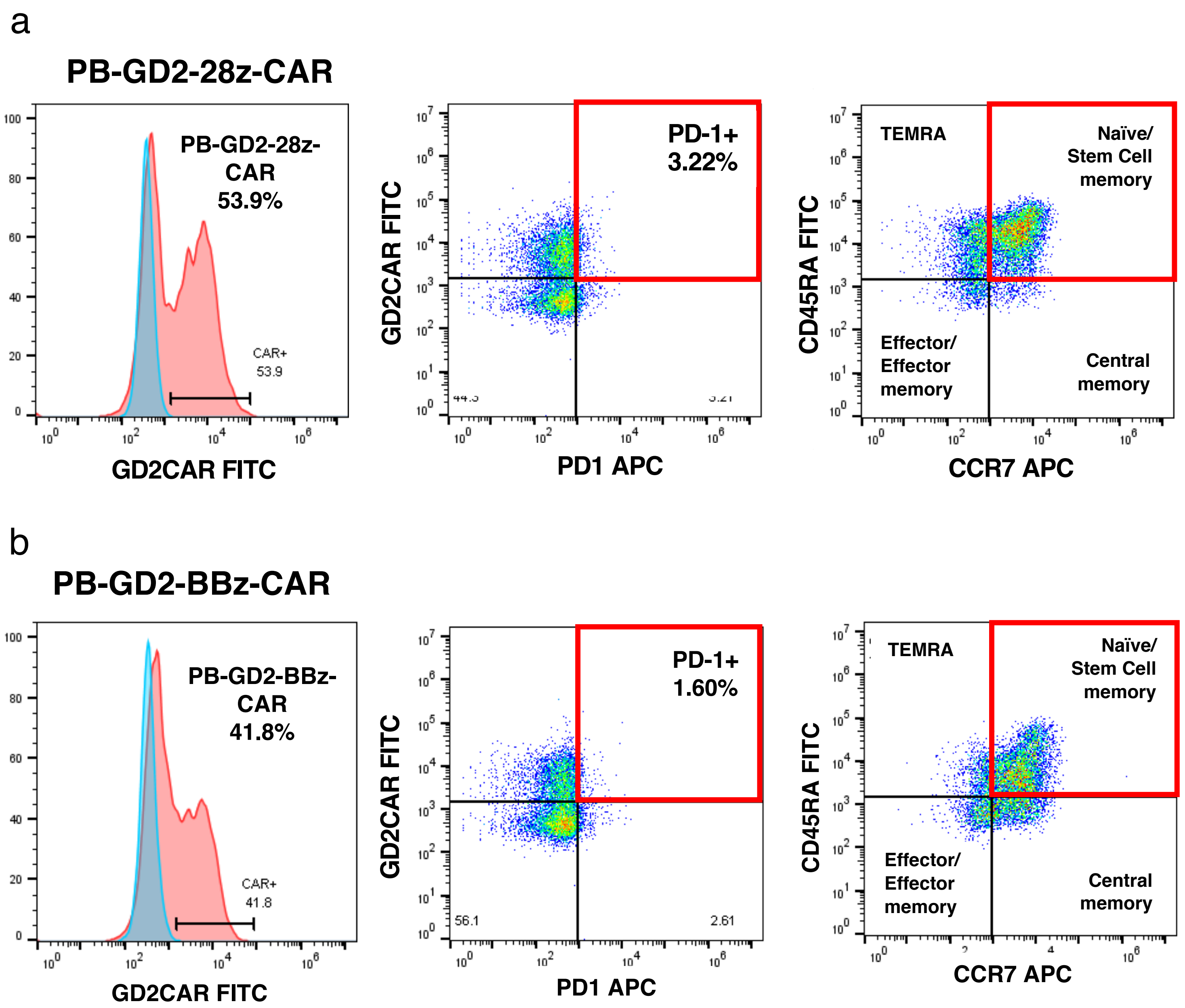


**Figure S8**
PB-GD2-CAR-T cells manufactured by CAR stimulating feeder cells. **a.** CAR expression and phenotype of PB-GD2-28z-CAR-T cells and **b.** GD2-BBz-CAR-T cells. GD2-28z, GD2-BBz transgene (Figure S1) were introduced into resting T cells on day 0, then co-cultured with CAR stimulating feeder cells expressing GD2/GD3 synthase together with CD80 and 4-1BBL, and cultured as described in Materials and Methods.

# Supplemental Figure S9

Corresponding Author; Shigeki Yagyu

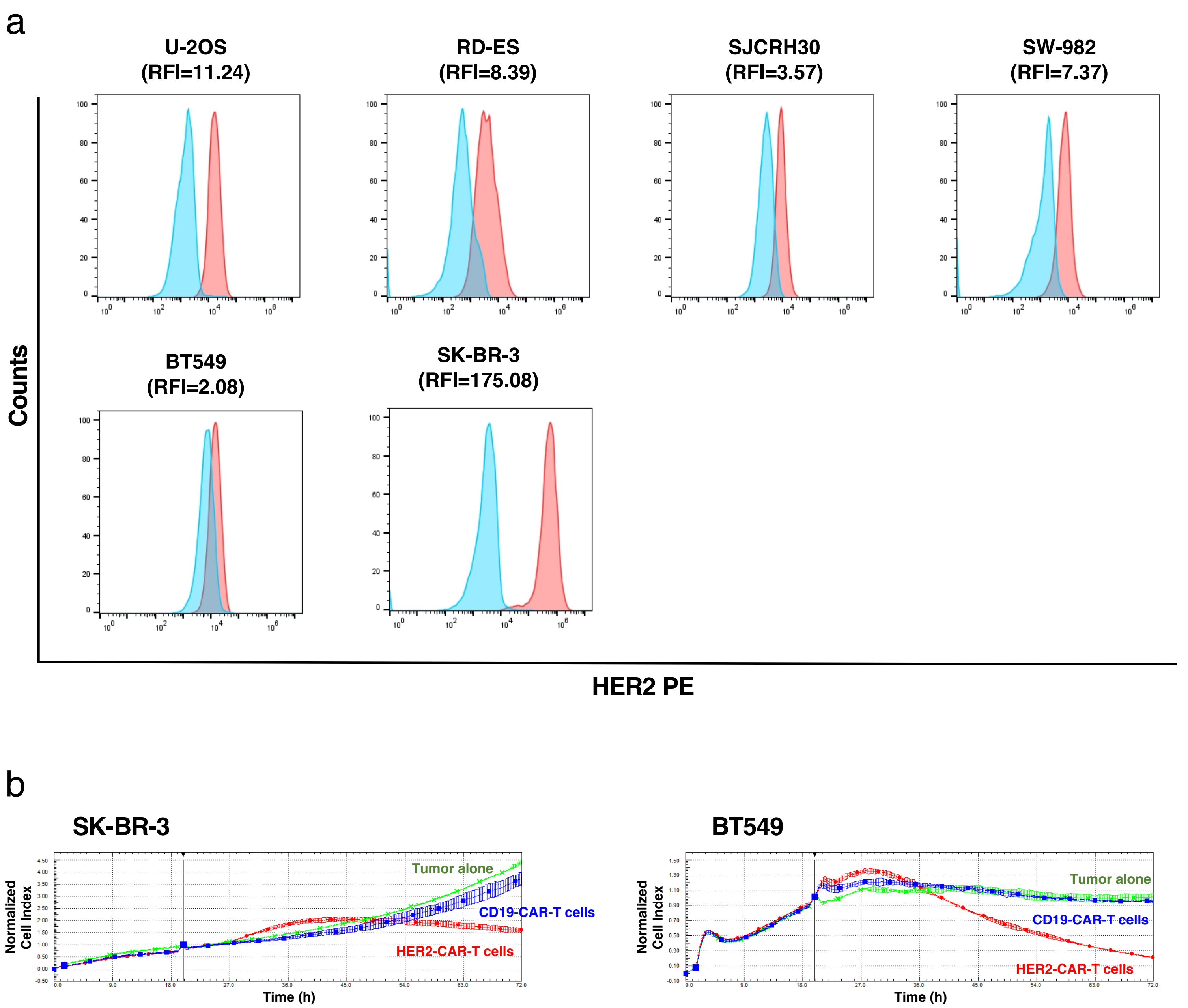


**Figure S9**

**a.** Expression of HER2 on various tumor cell lines determined by flow cytometry. **b.** Antitumor efficacy of PB-HER2-CAR-T cells against HER2-amplified (SK-BR-3) or triple negative (BT549) breast cancer cell lines. Real-time impedance traces of tumor cells without treatment (green) and tumor cells co-cultured with PB-CD19-CAR-T cells (blue) or PB-HER2-CAR-T cells (red) at an E:T ratio of 1:1 were acquired for 72 hours.

# Supplemental Figure S10

Corresponding Author; Shigeki Yagyu

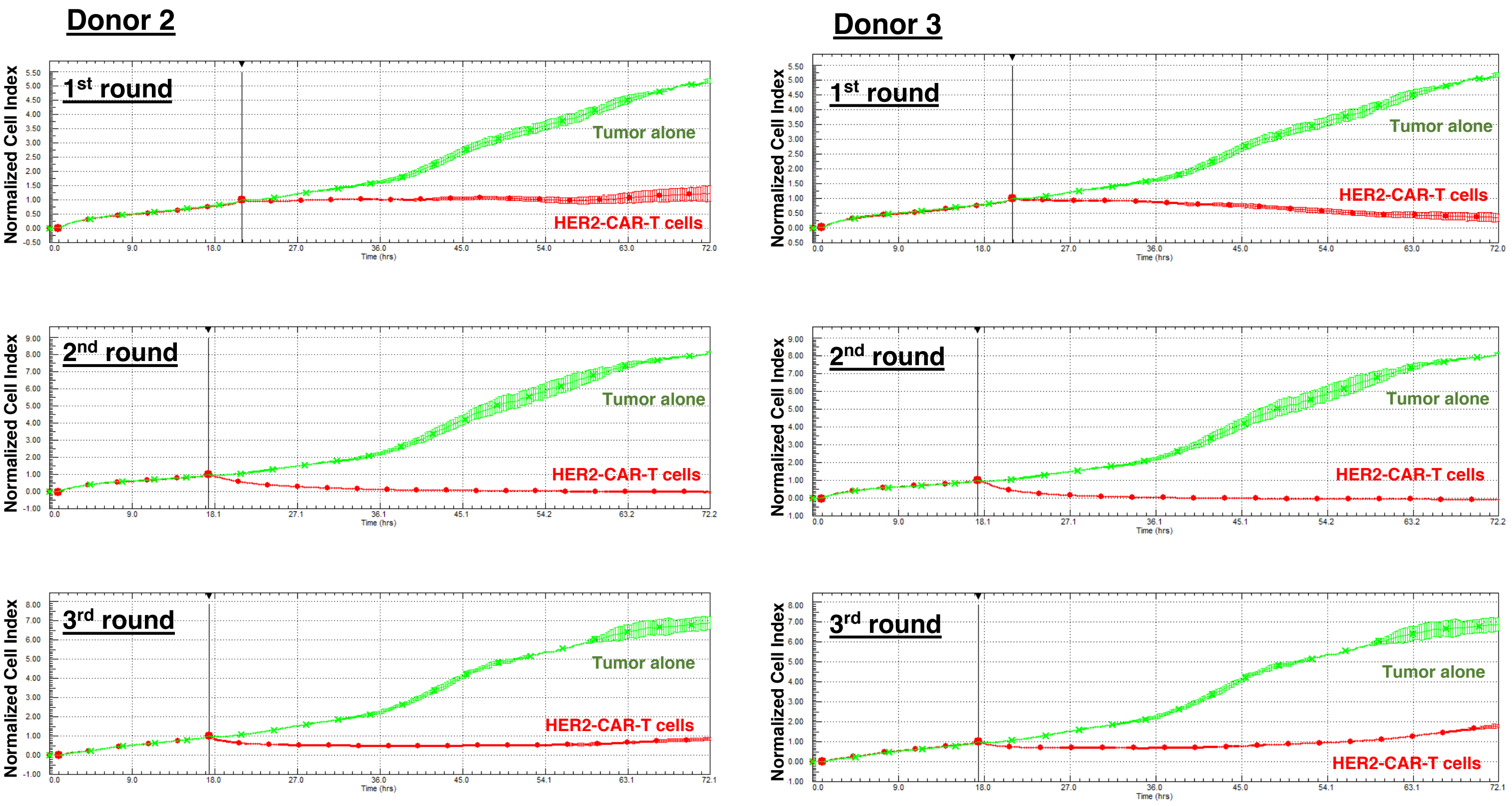


**Figure S10**
Sequential killing assay of PB-HER2-CAR-T cells from donor 2 and 3. Real-time impedance traces of tumor cells (green) and tumor cells co-cultured with PB-HER2-CAR-T cells (red) were acquired for 72 hours in each round.

# Supplemental Figure S11

Corresponding Author; Shigeki Yagyu

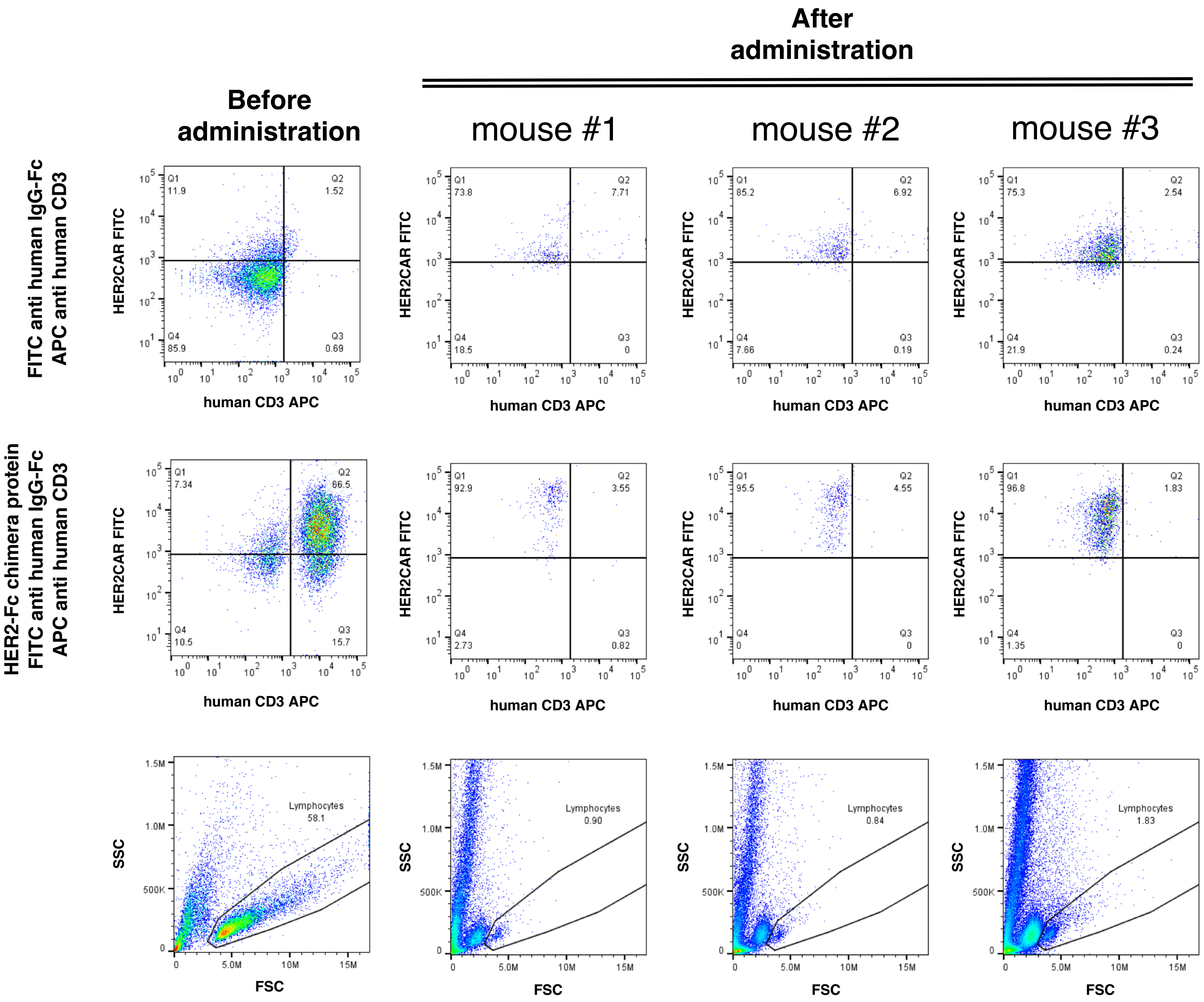


**Figure S11**

Non-specific binding of anti-human IgG-Fc antibody to mouse PBMC. PBMCs were isolated from untreated mice, then the PBMCs were stained with HER2-Fc chimera protein, anti-human IgG-Fc antibody, and anti-human CD3 antibody as described in Material and Methods. Mice PBMC (human CD3 negative population) were stained with anti-human IgG-Fc.

Corresponding Author; Shigeki Yagyu

**Supplemental Table 1**
List of antibodies used in this study

| Name | Clone/Accession # | Product# | Company |
|---|---|---|---|
| CD3-APC | UCHT1 | 300439 | BioLegend |
| CD3-BV421 | UCHT1 | 300434 | BioLegend |
| PD1-APC | EH12.2H7 | 329908 | BioLegend |
| PD1-BV605 | EH12.2H7 | 329924 | BioLegend |
| Tim3-APC | F38-2E2 | 345012 | BioLegend |
| LAG3-Alexa Fluor® 647 | 7H2C65 | 369204 | BioLegend |
| CD4-PE | RPA-T4 | 300508 | BioLegend |
| CD4-APC-Cy7 | RPA-T4 | 300518 | BioLegend |
| CD8-APC | HIT8a | 300912 | BioLegend |
| CD8-BV510 | RPA-T8 | 301048 | BioLegend |
| CD14-APC | TÜK4 | 130-113-705 | Miltenyi |
| CD19-APC | HIB19 | 302211 | BioLegend |
| CD45RA-PE | HI100 | 304108 | BioLegend |
| CD45RA-BV605 | HI100 | 304134 | BioLegend |
| CD56-APC | AF12-7H3 | 130-113-305 | |
| CCR7-APC | G043H7 | 353214 | BioLegend |
| CD28-PE | CD28.2 | 302907 | BioLegend |
| CD95-BV421 | DX2 | 305623 | BioLegend |
| HER2-PE | 24D2 | 324406 | BioLegend |
| CD80-PE | 2D10 | 305208 | BioLegend |
| 4-1BBL-APC | 5F4 | 311506 | BioLegend |
| Recombinant ErbB2/HER2-Fc | NP_004439 | 1120-ER | R&D SYSTEMS |
| Recombinant CD19-Fc | P15391 | 9269-CD | R&D SYSTEMS |
| Goat anti IgG-Fc-FITC | polyclonal | AP112F | Merck MIllipore |
